# No Major Differences Found between the Effects of Microwave-Based and Conventional Heat Treatment Methods on Two Different Liquid Foods

**DOI:** 10.1371/journal.pone.0053720

**Published:** 2013-01-16

**Authors:** Gábor Géczi, Márk Horváth, Tímea Kaszab, Gonzalo Garnacho Alemany

**Affiliations:** 1 Department of Environmental Engineering, Institute for Environmental Engineering Systems, Faculty of Mechanical Engineering, Szent István University, Gödöllő, Hungary; 2 Department of Chemistry and Biochemistry, Institute of Environmental Science, Faculty of Agricultural and Environmental Sciences, Szent István University, Gödöllő, Hungary; 3 Department of Physics and Control, Faculty of Food Science, Corvinus University of Budapest, Budapest, Hungary; 4 Escola Politècnica Superior, University of Vic, Vic, Spain; University of Central Florida, United States of America

## Abstract

Extension of shelf life and preservation of products are both very important for the food industry. However, just as with other processes, speed and higher manufacturing performance are also beneficial. Although microwave heating is utilized in a number of industrial processes, there are many unanswered questions about its effects on foods. Here we analyze whether the effects of microwave heating with continuous flow are equivalent to those of traditional heat transfer methods. In our study, the effects of heating of liquid foods by conventional and continuous flow microwave heating were studied. Among other properties, we compared the stability of the liquid foods between the two heat treatments. Our goal was to determine whether the continuous flow microwave heating and the conventional heating methods have the same effects on the liquid foods, and, therefore, whether microwave heat treatment can effectively replace conventional heat treatments. We have compared the colour, separation phenomena of the samples treated by different methods. For milk, we also monitored the total viable cell count, for orange juice, vitamin C contents in addition to the taste of the product by sensory analysis. The majority of the results indicate that the circulating coil microwave method used here is equivalent to the conventional heating method based on thermal conduction and convection. However, some results in the analysis of the milk samples show clear differences between heat transfer methods. According to our results, the colour parameters (lightness, red-green and blue-yellow values) of the microwave treated samples differed not only from the untreated control, but also from the traditional heat treated samples. The differences are visually undetectable, however, they become evident through analytical measurement with spectrophotometer. This finding suggests that besides thermal effects, microwave-based food treatment can alter product properties in other ways as well.

## Introduction

Microwave heat treatment of food products results in both thermal and non-thermal effects. The non-thermal effects are reactions and processes, during which the physical, chemical or biological conditions of the product change without an increase in its temperature.

The application of well-known heat transfer mechanisms (conduction, convection, radiation) and - by another classification - direct and indirect heating methods offer a number of possibilities for heat treatments of food products. For heat treatments of liquid food, the most frequently used methods are plate- or tube-based heat exchangers, where heat transfer is applied indirectly using water or steam.

The higher speed of the internal heating - compared to that of the traditional methods based on external heat transfer and heat conduction - made microwave equipment popular for these applications. Its thermal application is characterized by release of thermal energy inside the material being heated. One of the most significant benefits of microwave heating is reduced damage to nutritional value due to the shorter duration of the heat treatment [1], [2], [3], [4]. Additional advantages include energy savings, lower operating costs, fast processing and flexibility; all of which could make the use of microwave attractive in both industrial and small-scale applications [5], [6].

The application of industrial microwave heating using a microwave-based milk pasteurizer operating in continuous mode on 82.2°C was tested in the milk industry by Hamid and colleagues [7] to decrease the count of those bacteria that are responsible for spoiling food. Besides applying the microwave heat treatment successfully in a few areas of the food industry (e.g. sterilization, dehydration, leavening or baking of bread; for review see [8], [9]), the heat treatment of liquid foods has also been attempted. The main problem with liquid heating by microwave is that during heating by microwave the sterilization effect is not guaranteed due to uneven temperatures within the product. In addition, there is a view in the popular press that the use of microwave energy may have adverse effects. Although there are few properly conducted studies published in the peer-reviewed literature to support this view (see e.g. [10], [11], as examples), this view continues to exert an adverse effect; namely, inhibiting the spread of technology both in industry and households.

When the vitamin B1, B2 and B6 contents of milk were analyzed following treatments of microwave and traditional (tube heat exchanger) heating methods, it was found that neither of the two methods caused a decrease in these vitamins [3], [4]. Microwave heat treatment was shown to be useful for mild milk pasteurization [12]. We can also find additional examples for the analysis of the vitamin content in microwave heat-treated milk. For example, certain treatment combinations (e.g. 520W, for 4 minutes, ∼83°C or 500W, for 6 minutes) caused a higher degree of decomposition of vitamins A and B12, but in other aspects these publications also prove the advantages of the microwave and its applicability for heat treatment [13], [14].

The effects of microwave heat treatment on fruit juices have been studied at length as this process is often part of their manufacturing. The quality of citrus is determined by the enzyme reactions in the fruit not only in the growth phase, but during processing as well. For example, inactivating the methyl ester pectin is especially important in prolonging shelf life. Studies indicate that the pasteurization of fruit juices can be performed faster with smaller decrease in ascorbic acid at the same time. In case of citrus juices, the latter clearly determines freshness [2]. Microwave heat treatment, as a feasible alternative in food processing technology, significantly decreased the initial bacterial count of fruit [6]. It has also been proven that the decrease of the amount of mold (Aspergillus sp) is more substantial using microwave-based heat treatment [15].

Since it was proposed that microwave heat treatment may have other effects in addition to thermal ones, some researchers have been focusing on demonstrating these non-thermal effects. A number of authors have carried out low temperature enzyme inactivation with microwave irradiation to prove the existence of the non-thermal effects [16], [17], [18], [19]. However, data from Shazman suggests that non-thermal events due to microwave heating do not exist based on their large-scale experiments [20]. They studied the products of the Maillard reaction, the denaturation of proteins, as well as NaCl solubility after microwave treatment. They also performed mutagenicity tests. In contrast with earlier publications, the non-thermal effects did not occur significantly.

Neményi et al. prove using a variety of liquid foods that achieving homogeneous heating in an intermittent operating mode microwave electromagnetic field is difficult. Even heating is achieved using water traps and the effects of the microwave radiation on the behavior of the lipase and xanthine oxidase enzymes in milk are studied [21], [22]. Szerencsi et al. have established that the spreading of Saccharomyces cerevisiae cells was greater due to the microwave radiation, which can be attributed to its non-thermal effects [23]. Lakatos et al. have detected differences in the size of the milk fat globules treated with microwave in contrast to traditional pasteurization technologies [24]. Csapó et al. and Albert et al. developed a continuous mode microwave method for microwave pasteurization. Their findings conclude that, in contrast to convection technology, the microwave treatment may result in a higher degree of damage to vitamin C [25], [26].

We developed in-house instrumentation which enabled a comparative study of the effects of different heating methods. In this study we compare the effects of home-made circulating microwave heating to conventional heating with identical heating time and temperature with orange juice and milk as a models.

## Materials and Methods

### Ethics Statement

We have not performed any experiment on animals or humans. Although volunteers were asked to perform sensory tests, liquid foods identical or very similar to those that can be purchased from commercial sources were used in these tests, the general precautions of food safety were taken into consideration and volunteers were informed about these facts. Therefore, in agreement with the corresponding laws of the EU (852/2004/EK), such tests would not require special permissions.

### Orange Juice and Milk Samples

Orange juice samples were freshly squeezed from commercially available Navelina oranges originating from Seville (Spain) purchased at local supermarkets. For each test we used 13–15 kg orange to obtain 5 litres of orange juice. The orange juice was filtered using a coarse plastic filter of a Hauser brand household blender to remove the seeds and pulp and was then poured into 5-liter plastic jugs. The physical parameters, specifically the total acid content, vitamin C content, pH and the 24-hour sedimentation of the freshly squeezed orange juice varied in a wider range during the six-month duration of experiments than those of the concentrated orange juice samples (Table S1).

We also used non-pasteurized orange juice produced from concentrate provided by Gramex Ltd. (Veresegyház, Hungary). This juice required no preparation. The company provided the material for the experiments in 5-liter plastic bottles obtained from the production line after mixing, but before heat treatments.

Fresh milk samples were obtained from Új Mező Ltd. (Egyházasdengeleg, Hungary). After morning milking, filtering and cooling to 4°C, the samples were transported at <9°C to the experiment site. Filtering was performed directly after milking and before cooling using a paper filter with a 3.25 litre/min/cm2 filtration rate (DeLaval 620×60, 60 g/m2, Finland). According to test reports by Livestock Performance Testing Ltd. of Gödöllő, the milk samples arrived from the production site with 3.71±0.21g/100 g fat content and 3.32±0.14 g/100 g protein content. The total viable cell count varied between 50.000 and 350.000 CFU/cm^3^.

The samples were coded before performing the tests. Double-blind tests were performed: the person performing the analysis received mixed and coded samples and the person doing the treatments did not partake in analyzing the samples.

### Reference Measurement Configuration and the Methods of Heating

**Figure 1:**
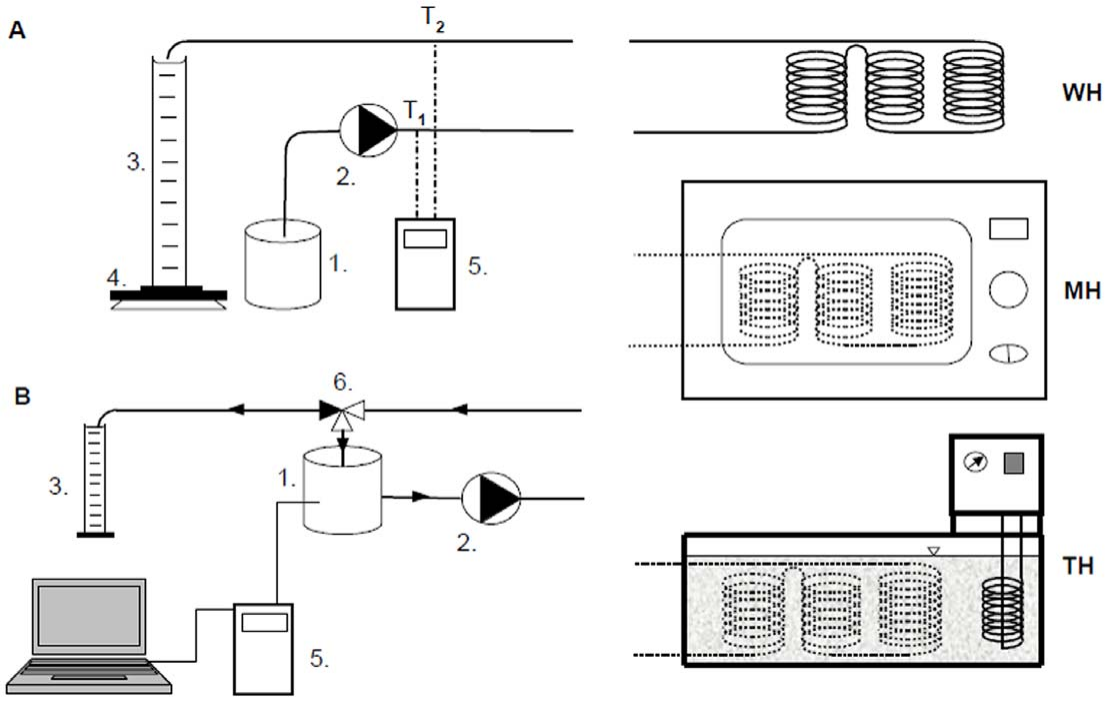
Modular experimental setup for heat-treatments of orange juice and milk. Panel A: flow-through heating; Panel B: heating with recirculation; MH – treated with microwave; TH – traditional heat treatment; WH – untreated control.

The test equipment was implemented by converting a household microwave oven into a flow-through, continuous operating mode device with 900 Watts output power. Two holes with seven mm diameter located 8 cm apart were made in the oven to introduce and drain the liquid. The microwave equipment complete with special glass spirals was connected to a STENNER 85M5 type adjustable feed rate peristaltic pump (Stenner Pump Company, Jacksonville, FL, USA), an XP-3000 type analytical scale (Denver Instrument GmbH., Gottingen, Germany) and an ALMEMO 2590-9 temperature measuring instrument (Ahlborn, Holzkirchen, Germany) (Panel A in Figure 1).

Inside the microwave oven, the liquid foods flowing through the glass spirals can be heated to the desired temperature depending on the length of the spiral and the flow rate of the peristaltic pump. The temperature can be easily monitored before entering and after leaving the microwave field, allowing the process to be controlled effectively. One of the advantages of this method is the gradual heating and constant output temperature due to the use of glass spirals. This way the temperature fluctuation characteristics of intermittent operation can be avoided. The gradual heating and the resulting temperature differences are presented in Figure S1.

To study the treatments where the milk/orange juice are heated in different ways, but under identical circumstances (i.e. the final temperature and the treatment time must be identical), the glass spiral implement was also used with a *T*-PHYWE type water bath (Lauda DR.R. Wobser GmbH, Lauda-Königshofen, Germany). By adjusting the water temperature, we were able to create the same treatment temperature as with the microwave method using an identical flow rate resulting in identical treatment time (Figure 1). This parallel process made it possible to compare milk/orange juice samples treated under identical circumstances, but with different heating methods. As control samples, milk and orange juice were passed through the glass spiral with no heating. As seen in Figure 1, the liquid foods were pumped through the the glass spirals with the peristaltic pump.

For each comparative test, the glass spirals were first placed into the Whirlpool AT 314 microwave oven (MH) and then into the *T*-PHYWE water bath thermostat (TH), respectively. The same glass spirals were used for the control samples (WH) without any heating. The temperature was continuously monitored before (T_1_) and after (T_2_) the treatment using the ALMEMO 2590-9 temperature measuring system. During each measurement, both (T_1_) and (T_2_) were kept constant. The treated and the control samples were fed into the storage containers according to the tests and their mass was measured using the Denver XP-3000 scale.

With the exception of the samples prepared for the ascorbic acid content test in the case of orange juice, we did not perform holding – the samples cooled down to the 8 to 10°C storage temperature naturally. The heating apparatus was supplemented with a three-way valve, which was used to mix the heated sample back to the storage container (see Panel B in Figure 1). By mixing the sample, we achieved the target temperature of 85°C (T_3_) in 13 minutes with both heating methods. Holding at the set temperature was achieved by continuing the flowing and heating for another 10 minutes before filling the liquid into the sample storage containers. During this test, the flow rate was set to Q = 1.19 cm^3^/s, and the temperature of the water bath thermostat was kept at 94.5°C (Figure S2).

### Storage Experiments

During the comparative storage experiments for both the milk and the orange juice we tested conditions that are uncharacteristic for each particular product. The samples were normally stored in a dark cellar, where the temperature varied between 8 to 10°C. The samples were continuously photographed using a Canon Powershot A430 camera. For the test under so-called accelerated conditions, we poured 20 g properly prepared orange juice into a Petri dish and stored it on open air at a room temperature of 21–23°C. The deterioration processes are accelerated when contacting air on a large surface. We repeated the observation four times with the freshly squeezed orange juice and three times with the concentrated orange juice.

To observe degradation of orange juice under normal storage experiments, 100 ml orange juice pre-treated in different ways was stored in polyethylene terephthalate (PET) bottles. Within this experiment, samples were divided into three additional treatment groups: open, not sealed; sealed; sealed and shaken daily. The storage temperature was 8–10°C. We conducted the test simultaneously on two sets of nine PET bottles. We repeated the tests three times with the freshly squeezed orange juice and twice with the concentrated orange juice. Regarding the changes during the storage experiments, we based our conclusions on the photos.

### Analysis of Food Properties

The comparative tests were performed on the experimental equipment assembled in the workshop of the Faculty of Mechanical Engineering at Szent István University. The analysis and testing of the orange juice and milk samples were conducted at the Department of Chemistry and Biochemistry, Szent István University and at the Department of Physics and Control, Faculty of Food Science, Corvinus University of Budapest. The vitamin C content and bacterial cell counts were performed by Livestock Performance Testing Ltd. After the normal storage tests, 12 ml gas samples were taken from the air above the orange juice by puncturing the plastic bottles. The gas samples were then injected into previously evacuated 12 ml volume special sampling tubes and were shipped to the laboratory to be directly analysed using a gas chromatograph (HP 5890 Serial II, USA). For the carbon-dioxide test, we utilized a TCD detector measuring thermal conductivity.

The colour properties of the milk and orange juice samples were determined using a ColorLite sph 850 spectrophotometer. Test results were obtained as CIE (Commission Internationale de la Éclargie) L*, a*, b* colour properties with wavelengths between 400 and 700 nm [27], [28], [29]. The settings of the instrument were the following: “2° standard observer” and “standard illuminant D65”. Results of each measurement were calculated from the average of three measurements by the ColorLite equipment. There are more than 6 million colour codes in the CIE Lab System. The colour parameters were the following: lightness - L*, which defines the grades of brightness from black to white, red-green colour coordinate - a* and yellow-blue colour coordinate - b* (Figure S3).

The colour parameters of the foods were monitored every 2–3 days throughout sample storage experiments for the orange juice. This required a large number of samples, as once the samples were placed into the spectrophotometer, they could not be used again. In case of orange juice samples, we prepared 15 aliquots of 25 ml sealed samples by sample groups (MH, TH and WH). From each group 3 samples were measured on 1^st^, 3^rd^, 6^th^, 8^th^ and 10^th^ day following the treatment.

The colour parameters of the milk samples were measured only on the day following the treatment. The cream forming during the storage of high-fat milk did not allow continuous, reliable colour measurement similar to that of the orange juice. We prepared 12 aliquots of 25 ml samples each from the three sample groups, which were tested on the day following the treatment. The visual examinations for milk were performed on five different days (on five different biological samples).

To determine the vitamin C content of the orange juice we asked the assistance of the aforementioned laboratory of Livestock Performance Testing Ltd, where the vitamin content of the beverages were measured using 2,6-dichlorophenolindophenol reaction with a titration method [30], [31]. We did not participate directly in these tests. We prepared 12 aliquots of 75 ml statistical samples from all five sample groups (WH, MH-A, TH-A, MH-B, TH-B) based on the pairs presented in Figure 1.

The taste tests of the orange juice were performed using a simplified procedure with the help of untrained volunteers (students and staff of the university), who did not participate in either the preparation or the experiments and analysis. We applied a triangle test, where the assessors’ task was to select the distinct third sample different form the other two [32], [33], [34]. If the assessor did not detected any difference between the samples, we asked them not to guess but mark the choice “CANNOT DETECT DIFFERENCE”. We’d like to emphasize that the participants were assured that one of the three samples is different from the other two identical ones. The purpose of this test was to establish if there was any difference between the taste of the treated and untreated sample and whether or not the two treatment methods can be distinguished based on the taste of the samples (Table S2– the arrangement of the triangle test).

Determination of bacterial counts, and quantification of protein and fat content of the milk samples were performed at the laboratories of Livestock Performance Testing Ltd. In order to determine the composition of the milk samples, an FT 6000 series Milko Scan spectrophotometer (Foss Electric, Denmark) was used. We created four daily statistical samples from each sample heated with the two different methods and the control sample (no heating) as well. The tests were performed a total of 17 times. The initial parameters of the tests were varied due to the nature of the procedure (fat content 3.71±0.21 g/100 g, protein content 3.32±0.14 g/100 g, total viable cell count 50.000–350.000 CFU/cm^3^). While creating the sample groups, the target temperature was kept constant during the day, but was varied between 64–82°C on each occasion. The target temperature was controlled by adjusting the flow rate or by replacing the spiral. To statistically demonstrate the decrease of the bacterial count we analysed two sample groups with a larger number of samples (16–16 and 24–24 parallels).

### Statistical Analyses

We calculated the statistical average of CO2 content, colour properties, vitamin C content and total viable cell count parameters of the samples groups created during the tests. To compare the averages of samples from the two heat treatment methods and the non-heated samples, we applied the Student’s two-sample *t*-test. We compared the total viable cell count decreasing effect of the heat treatments of milk, and in case of orange juice, the effect of the heat treatments on the vitamin C content.

When evaluating the taste comparison test of orange juice, we applied the laws of probability theory to decide whether or not the participants’ answers were given randomly. In some cases, such as when presenting the colour components or the increase in CO2 content during the storage of orange juice, we used basic descriptive statistical methods and the results are displayed on diagrams.

## Results

### There is no difference between the Beneficial Effects of Microwave and Traditional Heat Treatment on the Shelf Life, the Sedimentation of Fractions and the Generation of CO_2_ During the Storage of Orange Juice

**Figure 2:**
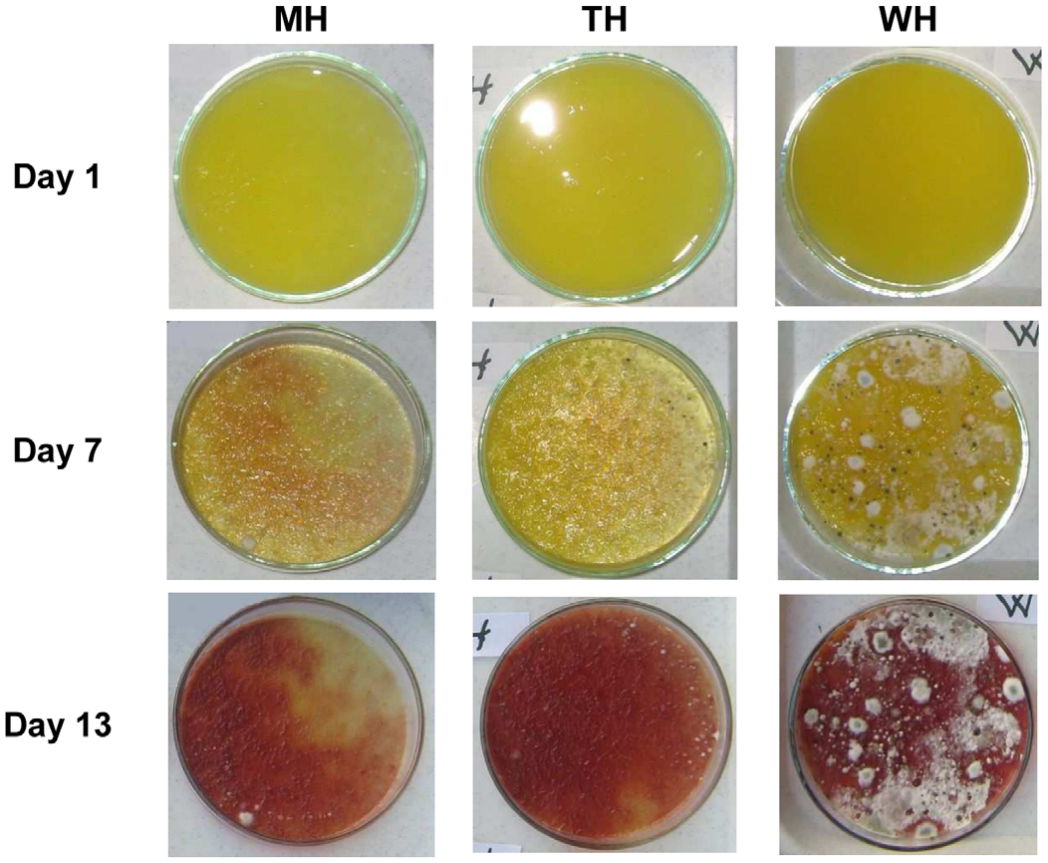
Both the microwave-based and the traditional heat treatment effectively delays surface deterioration of freshly squeezed orange juice samples at room temperature. MH – treated with microwave; TH – traditional heat treatment; WH – untreated control.

First, we demonstrate an example of storing orange juice at room temperature and in open air. There was no visible deterioration in either sample on the day following the creation of the samples and placing them in Petri dishes (Figure 2). The water content of the orange juice evaporated on the relatively large surface and the fibres dried out. On the untreated samples, intense moulding was visible even after one week, while on the heat-treated samples there was no mould or only traces of mould were visible (Figure 2). We did not observe any difference in changes during storage between the heat treated samples by visual survey.

After evaluating the sedimentation of the orange juice pulp in test tubes we concluded that the sedimentation of fraction slowed down compared to the untreated samples, but no differences were found between the samples treated with the two methods (data not shown).

**Figure 3:**
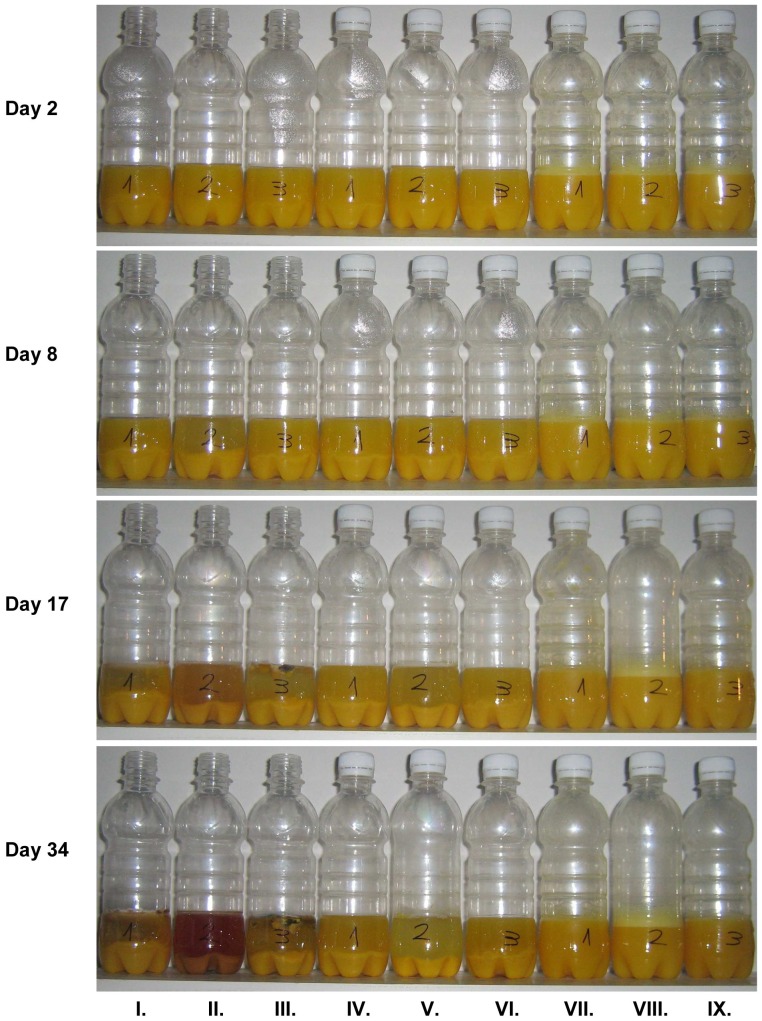
Storage of orange juice produced from concentrate in open containers, in sealed containers and in sealed containers shaken daily. Treatment groups of three: First 3 samples: open; second 3 samples: sealed and undisturbed; third 3 samples: sealed, shaken daily. Markings on the bottles: 1- microwave treated, 2- untreated control, 3- treated with water bath thermostat. The storage test was performed in two parallel experiments and each gave the results presented in the figure.

Figure 3 shows an example of the changes during storage of orange juice from concentrate under different conditions. Microwave-treated, traditional heat-treated (in the water bath thermostat) and untreated control samples were poured into PET bottles and were stored for 34 days in three different ways. The first three bottles (one from each treatment type) were left open, the next three were sealed, but left undisturbed, whereas the last three bottles were also sealed, but were shaken up daily throughout the whole period.

There were no visible difference in the colour of the samples on the second day, but we observed that the surface of the samples that were shaken was foamy (Figure 3, row day 2, VII., VIII., IX.). On the 8^th^ day the separation of fractions in the first two groups (I.-VI.) was already visible. Both on days 17 and 34, the untreated samples (marked ‘2′) in any of the three storage methods showed a difference in colour and foaming compared to the heat treated samples. The microwave- and water bath-treated samples stored under the same conditions indicated no visible differences.

**Figure 4:**
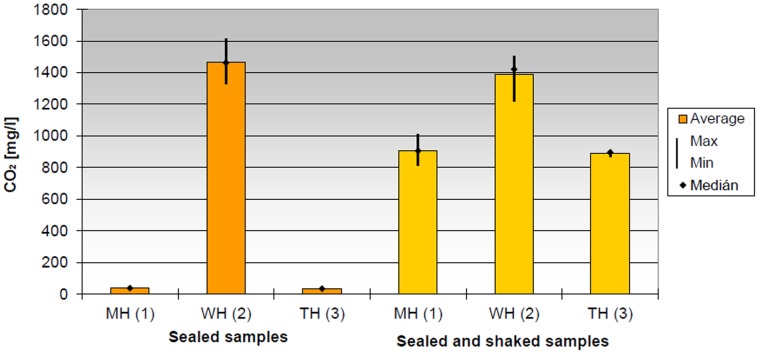
The CO2 content of gas in the bottles decreased similarly in microwave-treated and traditional heat-treated orange juice samples both in ‘closed’ and ‘closed and shaken’ situations. MH – treated with microwave; TH – traditional heat treatment; WH – untreated control. Each bar shows the average of six technical replicates.

Since the sealed bottles used for storing the untreated samples changed their shape (e.g. Figure 3, day 34, VIII.), samples were taken from the gas above the juice and were analysed using a gas chromatograph. We found that the gas above the control samples contained a relatively high amount of CO2 (approx. 1.4 g/L). The CO_2_ content of the undisturbed sealed samples was higher by the order of two magnitudes, while in case of the samples shaken daily was lower by 40% (Figure 4). The samples treated with different methods, but stored under the same conditions did not exhibit any differences.

### The Two Treatments do not Exert Any Significant Effect on the Colour, Vitamin C Content or Taste of Orange Juices

**Figure 5:**
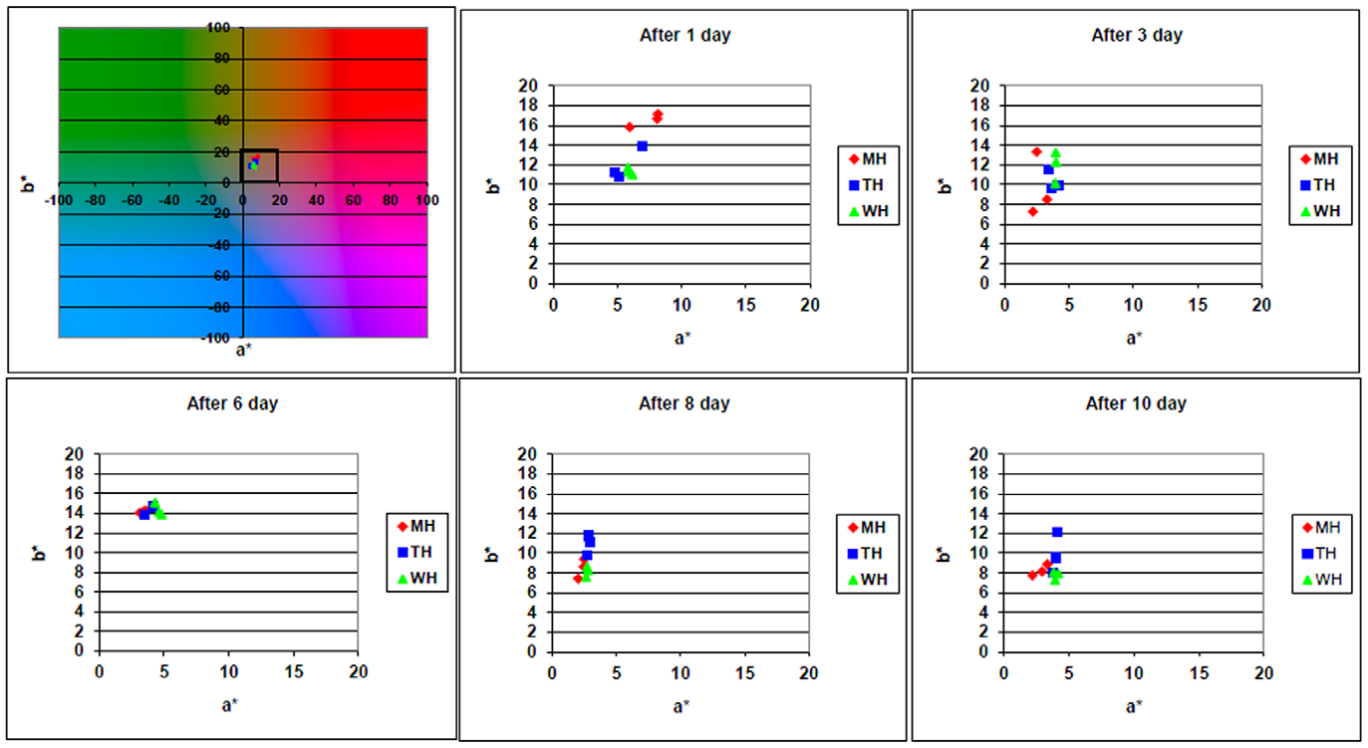
Colour parameters change of orange juice from concentrate in the period of 10 days following the treatment. The measured parameters: a* - red-green colour coordinate, b* - blue-yellow colour coordinate. MH – treated with microwave; TH – traditional heat treatment; WH – untreated control. (The L*-index of samples are shown in Figure 6.).

We also determined the colour of the product in CIE Lab system during the 10 days following the treatment for both the freshly squeezed orange juice and the orange juice form concentrate. We could not find any isolated cases across the sample groups (Figure 5), so neither the heat treatment itself, nor the method of treatment has a significant effect on the colour of the product in the early stages of storage. However, the photos of the previously presented storage experiments (Figure 3) clearly show that following the onset of deterioration, the untreated control group (WH) differs from the treated samples in colour as well.

**Figure 6:**
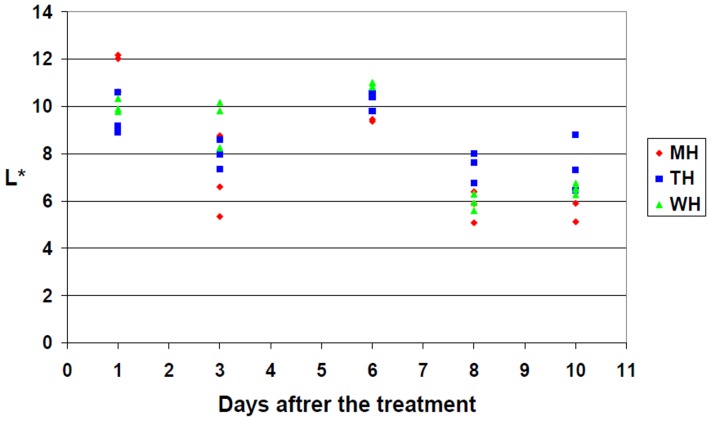
Lightness index (L*) change of orange juice from concentrate in the 10 days following the treatment. MH – treated with microwave; TH – traditional heat treatment; WH – untreated control. (The a* and b* of samples are shown in Figure 5.).

Besides the colour, the lightness of the product was also evaluated by determining the so-called L* lightness index. Although the lightness index of the orange juice from concentrate showed a slight change with time, the differences were not affected by the presence/absence or type of heat treatment (Figure 6). The test performed on freshly squeezed orange juice yielded similar results (data not shown).

**Figure 7:**
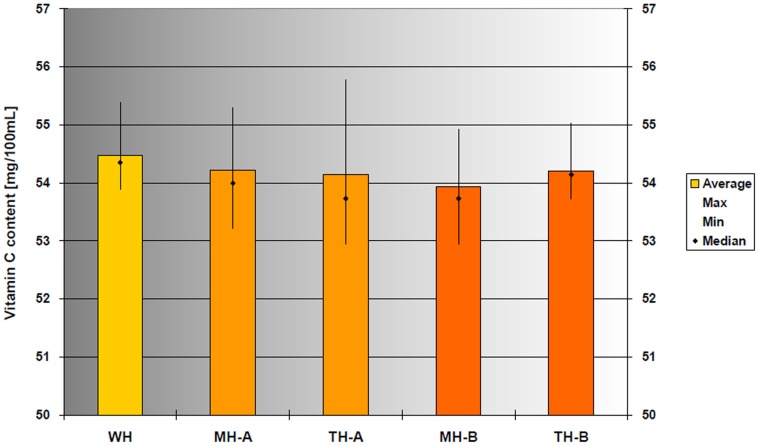
The vitamin C content of orange juice has not changed in microwave-treated or heat-treated samples at 85°C. WH: untreated control; MH-A: flow-through microwave treatment with no temperature holding; TH-A: flow-through traditional heat treatment with no temperature holding; MH-B: microwave heating of recirculated liquid; TH-B: traditional heating of recirculated liquid.

The change in vitamin C content due to heat treatment was also evaluated. Surprisingly, we found that the vitamin C content had not decreased during the heat treatment. Therefore, the experiments were repeated with both treatment methods with intense heating at 85°C followed by holding on temperature. Following treatment, we requested the analysis of the vitamin C contents of the samples from an accredited laboratory. The results clearly indicated that neither the microwave, nor the traditional heat treatment method caused significant decrease in vitamin C content compared to the control group (Figure 7). Statistical analysis (unequal variance paired t-test) performed on data from 12 replicates from one major batch of milk samples did not indicate significant difference in the probability variables (range: 0.06–0.38), indicating lack of difference between samples treated with different methods under different conditions (Table 1).

**Table 1:** Statistics for evaluating the decrease in vitamin C contents [mg/100 ml] in orange juice due to heat treatment.

No.	WH	MH-A	TH-A	MH-B	TH-B
1	53.89	53.81	55.78	53.73	53.73
2	54.31	53.21	54.93	53.73	55.03
3	54.31	53.93	53.73	53.73	54.39
4	54.31	55.29	53.73	52.95	54.39
5	55.03	53.81	53.73	53.73	54.81
6	54.39	54.22	54.93	52.95	54.39
7	55.03	54.06	52.95	54.93	53.89
8	55.39	53.21	53.73	53.73	53.89
9	54.31	55.29	52.95	54.93	53.73
10	53.89	53.21	53.73	52.95	53.73
11	54.39	55.29	55.78	54.93	54.81
12	54.39	55.29	53.73	54.93	53.73
	**WH**	**MH-A**	**TH-A**	**MH-B**	**TH-B**
Average	54.47	54.22	54.14	53.94	54.21
Max	55.39	55.29	55.78	54.93	55.03
Min	53.89	53.21	52.95	52.95	53.73
Median	54.35	54.00	53.73	53.73	54.14
SD	0.45	0.86	0.98	0.80	0.49
t_sz_ value		0.898	1.057	2.010	1.352
P(T< = t) two-tailed		0.38	0.31	0.06	0.19
t_p_ critical two-tailed		2.11	2.12	2.11	2.07
Result		|t_sz_|<t_p_	|t_sz_|<t_p_	|t_sz_|<t_p_	|t_sz_|<t_p_

WH: untreated control; MH-A: flow-through (Q = 1.49 cm^3^/s) microwave treatment with no temperature holding; TH-A: flow-through (Q = 1.49 cm^3^/s) traditional heat treatment with no temperature holding; MH-B: microwave heating of recirculated liquid (Q = 1.19 cm^3^/s); TH-B: traditional heating of recirculated liquid (Q = 1.19 cm^3^/s).

t – value of t-test to compare the averages of the two sample groups.

sz(index) – value calculated from the dataset.

p(index) – lookup value for a significance level of p  = 0,05.

The purpose of the sensory analyses was to find out whether the participants were able to distinguish on one hand the heat-treated and untreated orange juice samples and on the other hand the microwave-heated and water bath thermostat-heated samples based on taste. The test results are shown in Table 2.

**Table 2:** Test results for comparison of taste of heat treated and untreated orange juice.

Test	Number ofparticipants [no.]	Did not sense anydifference [no.]	Sense somedifference [no.]	Proper marking[no.]	Improper marking 1[no.]	Improper marking 2[no.]
1	41	4	37	19	8	10
2	41	3	38	22	9	7
1–2*		2*	36*	14*		
3	43	12	31	11	8	12
4	43	14	29	11	9	9
3–4*		10*	27*	4*		

* The two test series evaluated together.

In the test comparing the taste of the treated and untreated orange juice, most of the participants (MH vs. WH: 37/41; and TH vs. WH: 38/41) tasted some difference between the heat-treated and untreated samples. More than half of these respondents marked the correct answer in both cases. The difference was even more pronounced when evaluating the answers given to the two series together. We found that out of the 36 respondents who tasted a difference in both series, 14 persons selected the samples correctly, giving a hit rate of 38.9% as opposed to the 10.9% of statistical randomness.

In tests no. 3 and 4, the samples treated with water bath thermostat and microwave were compared. More than a quarter of the respondents did not sense any difference in the taste of the samples (test series 3 12/43, test series 4 14/43) and in both series only 11 persons could find the different sample. When evaluating the answers in series 3 and 4 together, we can conclude that 10 persons (or 23.2% of the respondents) could not sense any difference in either test. When considering the 27 test reports where the respondents clearly marked the different samples, there were only four persons who recognised the sample that was truly different. This translates to a hit rate of 14.8%, which is more than the statistical randomness, but the difference in not convincing enough.

Based on the test results, we can conclude that the participants were able to differentiate between the heat treated and untreated samples by sensory test, but they could not clearly distinguish between the heat treatment methods. The results presented refer to the freshly squeezed orange juice, but by performing the test on the orange juice concentrate products with fewer participants, we obtained similar results (data not shown).

### The Two Treatments Affect the Bacterial Count and Phase separation of Milk Samples in a Similar Manner

**Figure 8:**
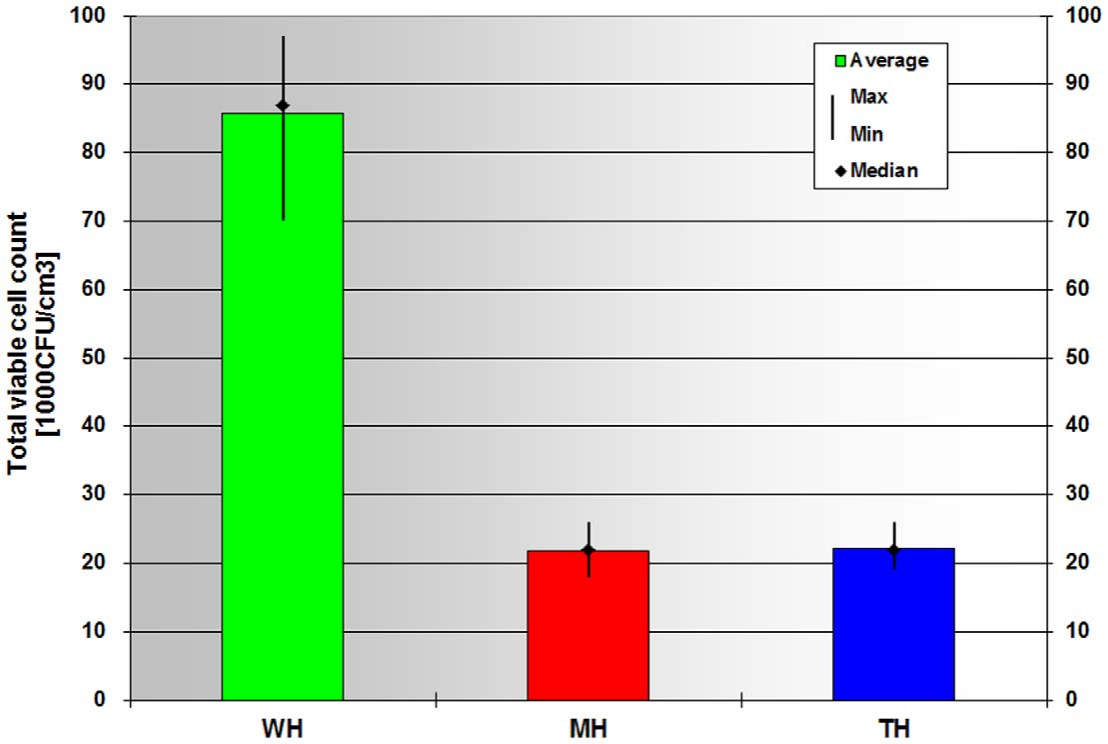
The total viable cell count in freshly milked milk significantly decreased after either of the two heat treatment methods. The average, maximum, minimum and median values of 24 statistical samples are shown per sample group.

The fresh milk was heat treated in the 64–82°C range with microwave energy transfer and water bath thermostat, then its bacterial count was evaluated by comparing it to that of the untreated sample. In 17 parallel tests lasting for four months a total of 85 litres of milk was treated and prepared for measurement. We provided 140 samples (100 ml each) to determine the total viable cell count. Both heat treatments resulted in a statistically significant decrease in the total viable cell count compared to control (P less than 0.0001, paired t-test): heating the fresh milk to 74.1±0.2°C (without holding on temperature) resulted in a 74.3% loss of the total viable cell count (Figure 8). There was no significant difference between the values obtained with the two different heating methods (Figure 8 and Figure S4; see also Table S3 for a representative example of the calculations). The laboratory tests also proved that the protein and fat content of the milk was unaffected by the heating (data not shown).

Similarly to the orange juice tests, the milk samples were stored under various conditions and were photographed continuously. There was a difference in amount of the separated milk fat, as well as the amount and colour of whey between the heat-treated and untreated samples (data not shown). However, similar to the above, no noticeable differences could be observed between the samples subjected to the two different heat treatment methods.

### The Colour of Microwave-treated Samples Differed not only from the Untreated Control, but also from Traditional Heat-treated Ones

**Figure 9:**
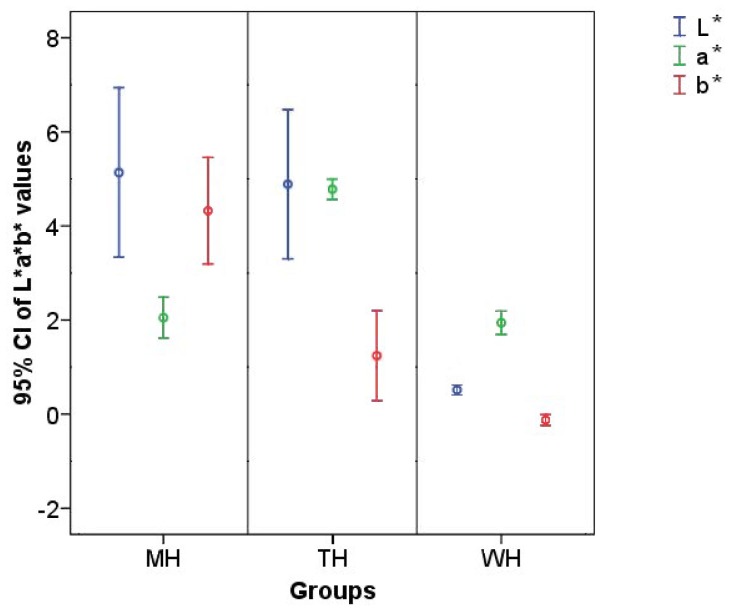
The colour parameters of milk were different after microwave treated vs. traditionally heat-treated samples. The error bar shows the 95% confidence interval (CI) of L*a*b* values at the three groups. The measured parameters: a* - red-green colour coordinate, b* - blue-yellow colour coordinate, L*- lightness index. The results of 12 statistical samples are shown by sample group.

The colour of the milk samples was evaluated with the same procedure used for the orange juice. In case of milk, the colour changes during storage were not monitored, but the effects of heat treatment on the colour of the products were analysed. According to our results, the colour of the microwave treated samples differed not only from the untreated control, but from the traditional heat treated samples as well (Figure 9). This experiment was repeated on milk samples obtained on 5 different days, and we observed the deviation in the colour and brightness of the samples treated with the two different methods in four cases. During the repetitions, the target temperature was varied between 72°C and 83°C. According to our results, the initial bacterial count, the fat content and the target temperature did not affect the separation of colour parameters. For the tested samples the average and the 95% confidence interval of the L*a*b* values versus handling methods are demonstrated in Figure 9. There were not significant differences in L*a* values between the groups of milk samples. However the b* values show significant difference between the groups. Furthermore, the L* and a* values were not significantly different between the MH and TH groups. Finally, the standard deviations of the L*, a*, b* values in these groups are bigger than the values of WH group.

## Discussion

The microwave-based heat treatment method produces uneven heating in the product due to the inhomogeneity of the electromagnetic field. By continuously transferring liquid foods through a microwave field, a specific heating can be achieved depending on the length of the glass spiral and the flow rate of the metering pump. Identical heating levels and intensity can also be achieved by transferring the liquid through a water bath instead of microwave field. Adjusting the temperature of the water bath affects the results, but using this parallel method microwave energy transfer and heat treatment based on convective heat transfer can be compared through the properties of the treated food product.

Orange juice samples treated with microwave and traditional heat-based methods as well as untreated controls were stored for the long term under different conditions (open, sealed-undisturbed and sealed-shaken). Based on the carbon-dioxide content of the air samples taken from the bottles, both treatment methods slowed down the fermentation processes in orange juice in comparison to untreated controls. The deterioration processes and the separation of the fractions in the undisturbed juices were both delayed as well. For each of the above listed parameters, no differences were detected between the effects of the two different heat treatment methods based on the photos taken during the experiments.

We utilised continuous colour monitoring during the storage of the orange juice. The Lab colour properties of all three control groups (WH, TH, MH) changed equally during the first 10 days of storage. Again, the two different treatment methods did not cause different effects. Interestingly, no negative effects of the heat treatment methods were detected when evaluating the vitamin C content. The vitamin C content decreased neither with the flow-through heating, nor with heated volume back mixing orange juice held on temperature for 10 minutes. The target temperature was 85°C in both cases.

The beneficial effects of the heat treatments were obvious when analysing the total viable cell count in the milk samples. We performed heat treatments 17 times at a minimum of 64°C and a maximum of 82°C without holding on temperature. We found that the decrease in total viable cell count was identical with both the microwave heat transfer and the water bath heat treatment. In our studies, differences of the effects of the heat treatment methods were detected only in the Lab colour characteristics of the milk samples, both in terms of the colour coordinates and the brightness index. The variation amounts to four units on a scale of 100, that is invisible to the naked eye. However, being able to detect a difference encourages us to continue our studies. To answer the question posed in the abstract based on the test conducted, we consider the microwave heating equivalent, but non-thermal effects cannot be ruled out.

Our studies might provide proof for food processing facilities that the whole volume of liquid products can be heated up in a microwave in a homogeneous manner.

## Supporting Information

Figure S1 **Flow-through microwave equipment with thermal images demonstrating the gradual heating (on right) and the temperature difference (on left).**
(TIF)

Figure S2 **The temperature measured in the container for the mix-back heating method versus elapsed time.** MH – treated with microwave; TH – traditional heat treatment; WH – untreated control.
(TIF)

Figure S3 **Interpretation of the CIELab system colour properties** [**29**] **.**
(TIF)

Figure S4 **Decrease of the total viable cell count in fresh milk according to the treatment method.** Samples were evaluated daily throughout a period of four days using four technical replicates per sample group. The temperature of the treatment: 08/02/2011–70.5±0,2°C; 09/02/2011–73.8±0,3°C; 10/02/2011–64.7±0,2°C; 11/02/2011–71.5±0,1°C.
(TIF)

Table S1 **Selected nutritional values and properties of the orange juices tested.**
(DOCX)

Table S2 **Triangle test configuration for comparing the taste of heat-treated and untreated orange juice.**
(DOCX)

Table S3 **Decrease in total viable cell count due to heat treatment in fresh milk.**
(DOCX)
