# Supplementary figures and images for: No Major Differences Found between the Effects of Microwave-Based and Conventional Heat Treatment Methods on Two Different Liquid Foods
Source: PLoS One. 2013 Jan 16;8(1):e53720. doi: 10.1371/journal.pone.0053720 (PMC3547058; doi:10.1371/journal.pone.0053720)

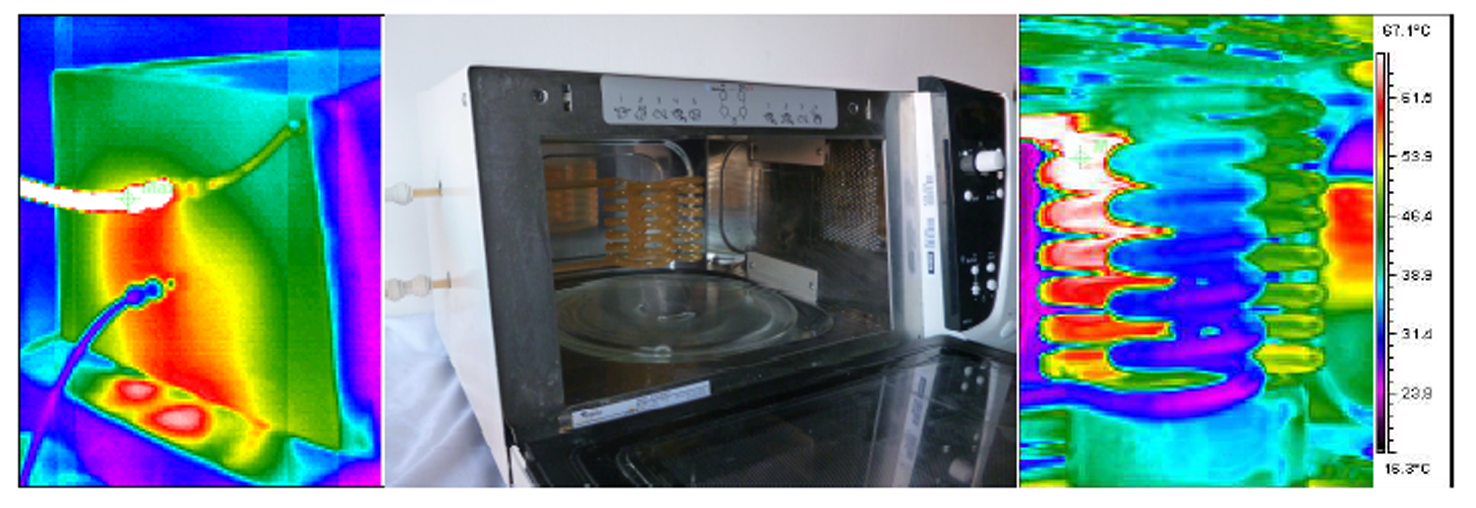

Supplement: Figure S1 — Flow-through microwave equipment with thermal images demonstrating the gradual heating (on right) and the temperature difference (on left). (TIF) [file pone.0053720.s001.tif]

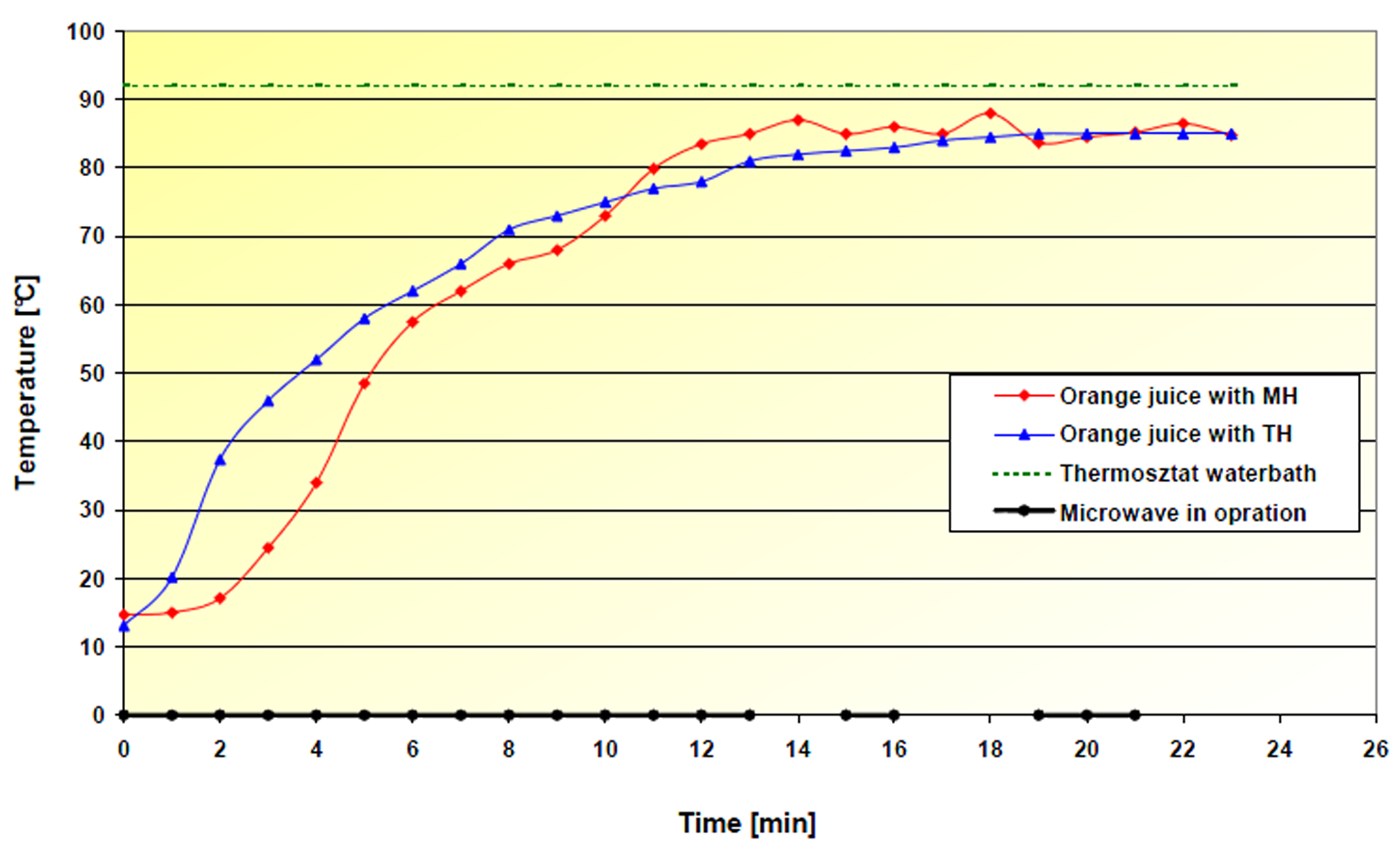

Supplement: Figure S2 — The temperature measured in the container for the mix-back heating method versus elapsed time. MH – treated with microwave; TH – traditional heat treatment; WH – untreated control. (TIF) [file pone.0053720.s002.tif]

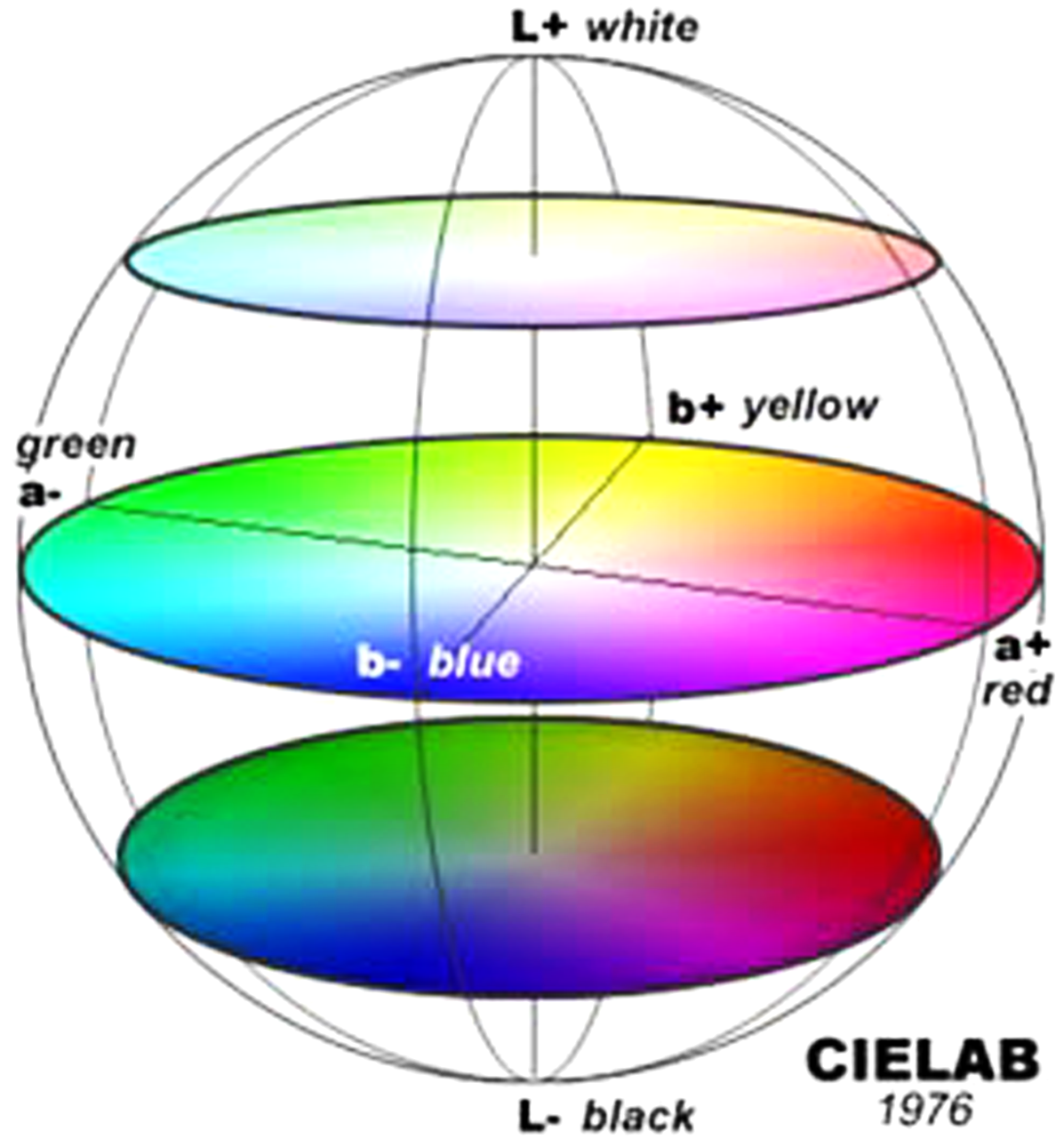

Supplement: Figure S3 — Interpretation of the CIELab system colour properties [29] . (TIF) [file pone.0053720.s003.tif]

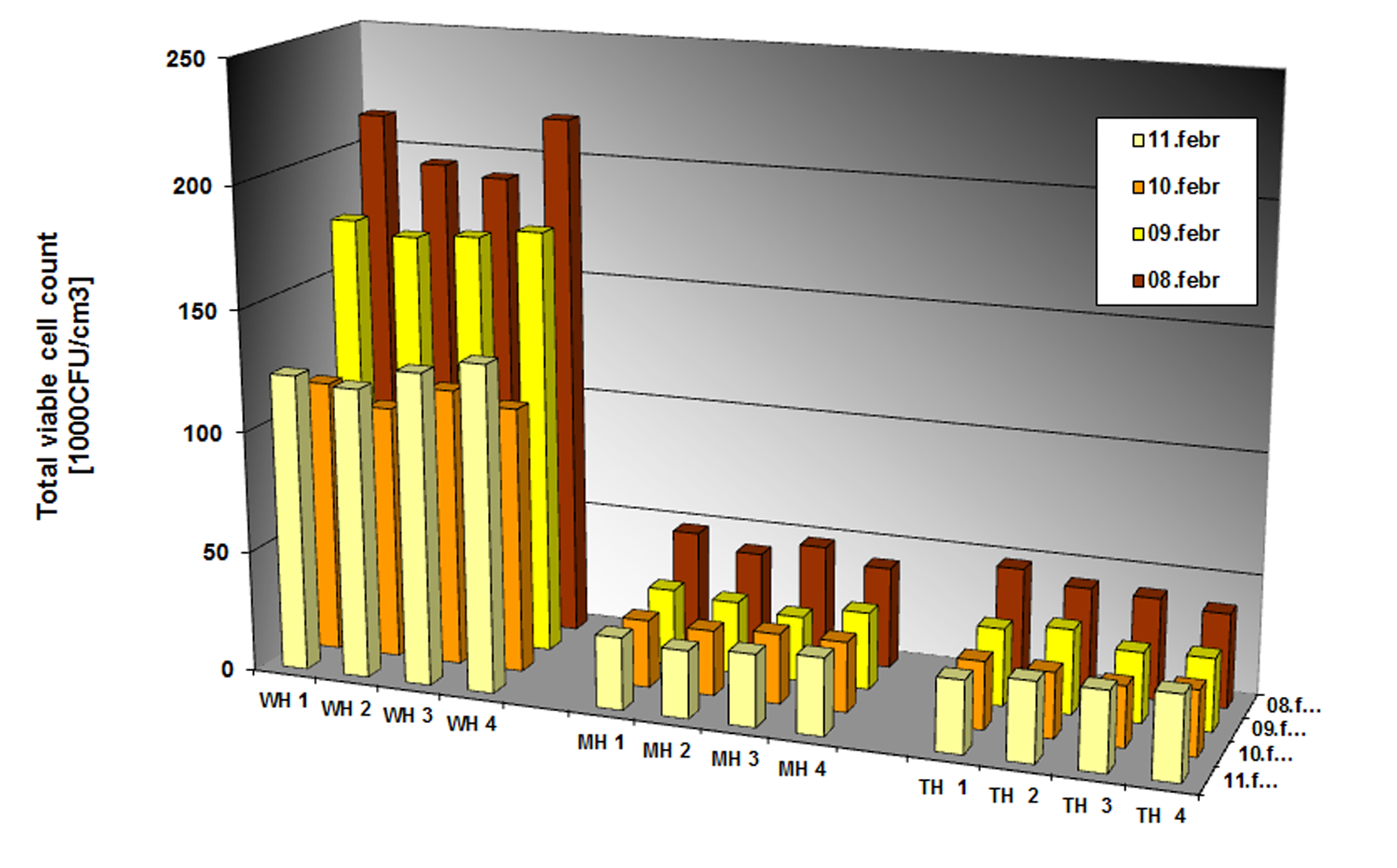

Supplement: Figure S4 — Decrease of the total viable cell count in fresh milk according to the treatment method. Samples were evaluated daily throughout a period of four days using four technical replicates per sample group. The temperature of the treatment: 08/02/2011–70.5±0,2°C; 09/02/2011–73.8±0,3°C; 10/02/2011–64.7±0,2°C; 11/02/2011–71.5±0,1°C. (TIF) [file pone.0053720.s004.tif]
