# Supplementary material for: No Major Differences Found between the Effects of Microwave-Based and Conventional Heat Treatment Methods on Two Different Liquid Foods
Source: PLoS One. 2013 Jan 16;8(1):e53720. doi: 10.1371/journal.pone.0053720 (PMC3547058; doi:10.1371/journal.pone.0053720)
Supplement: Table S1 — Selected nutritional values and properties of the orange juices tested. (DOCX) [file pone.0053720.s005.docx]

**Table S1. Selected nutritional values and properties of the orange juices tested.**

|  | Freshly squeezed  orange juice | Orange juice  from concentrate |
| --- | --- | --- |
| Dry matter content [%] | 10.34±0.02 | 11.38±0.04 |
| Sugar content [mass concentration %]* | 38.1±2.21 | 47.62±4.22 |
| Total acid content [mass concentration %]** | 1.19±0.04 | 0.90±0.02 |
| vitamin C content [mg/100ml] | 55.43±3.45 | 43.22±1.87 |
| pH | 3.43-3.85 | 3.76-3.79 |
| Density [g/cm^3^] | 1.004±0.005 | 0.984±0.007 |
| 24-hour sedimentation [%]*** | 37.06±2.61 | 17.86±1.55 |
| Light absorption (absorbance)**** | 0.197±0.012 | 0.225±0.024 |

*per glucose unit

**as tartaric acid equivalent

***volumetric proportion of settled fractions

**** on a wavelength of λ = 450 nm
