# Supplementary material for: No Major Differences Found between the Effects of Microwave-Based and Conventional Heat Treatment Methods on Two Different Liquid Foods
Source: PLoS One. 2013 Jan 16;8(1):e53720. doi: 10.1371/journal.pone.0053720 (PMC3547058; doi:10.1371/journal.pone.0053720)
Supplement: Table S2 — Triangle test configuration for comparing the taste of heat-treated and untreated orange juice. (DOCX) [file pone.0053720.s006.docx]

**Table S2. Triangle test configuration for comparing the taste of heat-treated and untreated orange juice.**

| **Test** | **Sample types** | | |
| --- | --- | --- | --- |
| 1 | WH | MH | WH |
| 2 | WH | WH | TH |
| 3 | MH | TH | MH |
| 4 | MH | TH | TH |

MH – treated with microwave; TH – traditional heat treatment; WH – untreated control.
