# Supplementary material for: No Major Differences Found between the Effects of Microwave-Based and Conventional Heat Treatment Methods on Two Different Liquid Foods
Source: PLoS One. 2013 Jan 16;8(1):e53720. doi: 10.1371/journal.pone.0053720 (PMC3547058; doi:10.1371/journal.pone.0053720)
Supplement: Table S3 — Decrease in total viable cell count due to heat treatment in fresh milk. (DOCX) [file pone.0053720.s007.docx]

**Table S3. Decrease in total viable cell count due to heat treatment in fresh milk.**

|  | *Total viable cell count*  *[1000CFU/cm^3^]****^i^*** | | |
| --- | --- | --- | --- |
| No. | WH | MH | TH |
| 1 | 70 | 18 | 24 |
| 2 | 85 | 20 | 22 |
| 3 | 90 | 19 | 24 |
| 4 | 89 | 24 | 24 |
| 5 | 97 | 22 | 24 |
| 6 | 75 | 24 | 19 |
| 7 | 85 | 24 | 22 |
| 8 | 75 | 26 | 20 |
| 9 | 96 | 22 | 19 |
| 10 | 92 | 20 | 24 |
| 11 | 96 | 19 | 22 |
| 12 | 93 | 24 | 24 |
| 13 | 89 | 22 | 24 |
| 14 | 79 | 24 | 26 |
| 15 | 78 | 19 | 21 |
| 16 | 87 | 19 | 20 |
| 17 | 78 | 24 | 24 |
| 18 | 87 | 22 | 19 |
| 19 | 78 | 24 | 19 |
| 20 | 93 | 19 | 24 |
| 21 | 92 | 24 | 22 |
| 22 | 88 | 18 | 24 |
| 23 | 85 | 22 | 20 |
| 24 | 79 | 24 | 21 |
| Expected value | | 21.79 | 22.17 |
| Variance | | 5.91 | 4.58 |
| F_sz_ value | | 1.291 | |
| F_p_ value | | 2.014 | |
| t_sz_ value | | -0.567 | |
| t_p_ value | | 2.013 | |
| **Result** | | \|t_sz_\| < t_p_ | |

F – The value of F-test for comparing the deviation of the two sample groups

t – The value of the t-test for comparing the average of the two sample groups

sz(index) – calculated value from the dataset

p(index) – lookup value for a significance level of p < 0,05
